# Preparative Scale Production of Recombinant Human Transthyretin for Biophysical Studies of Protein-Ligand and Protein-Protein Interactions

**DOI:** 10.3390/ijms21249640

**Published:** 2020-12-17

**Authors:** Ellen Y. Cotrina, Marta Vilà, Joan Nieto, Gemma Arsequell, Antoni Planas

**Affiliations:** 1 Laboratory of Biochemistry, Institut Químic de Sarrià, Universitat Ramon Llull, 08017 Barcelona, Spain; eycc7.lost@gmail.com (E.Y.C.); martavila87@gmail.com (M.V.); z_ener@hotmail.com (J.N.); 2 Institut de Química Avançada de Catalunya, Consejo Superior de Investigaciones Científicas (IQAC-CSIC), 08034 Barcelona, Spain; gemma.arsequell@iqac.csic.es

**Keywords:** transthyretin, recombinant expression, fed-batch culture, protein yield, protein-ligand interactions, protein-protein interactions, amyloid diseases

## Abstract

Human transthyretin (hTTR), a serum protein with a main role in transporting thyroid hormones and retinol through binding to the retinol-binding protein, is an amyloidogenic protein involved in familial amyloidotic polyneuropathy (FAP), familial amyloidotic cardiomyopathy, and central nervous system selective amyloidosis. hTTR also has a neuroprotective role in Alzheimer disease, being the major Aβ binding protein in human cerebrospinal fluid (CSF) that prevents amyloid-β (Aβ) aggregation with consequent abrogation of toxicity. Here we report an optimized preparative expression and purification protocol of hTTR (wt and amyloidogenic mutants) for in vitro screening assays of TTR ligands acting as amyloidogenesis inhibitors or acting as molecular chaperones to enhance the TTR:Aβ interaction. Preparative yields were up to 660 mg of homogenous protein per L of culture in fed-batch bioreactor. The recombinant wt protein is mainly unmodified at Cys10, the single cysteine in the protein sequence, whereas the highly amyloidogenic Y78F variant renders mainly the *S*-glutathionated form, which has essentially the same amyloidogenic behavior than the reduced protein with free Cys10. The TTR production protocol has shown inter-batch reproducibility of expression and protein quality for in vitro screening assays.

## 1. Introduction

The availability of large amounts of proteins that have potential biomedical applications is often limited in the initial steps of research. Particularly demanding is the setup of in vitro screening programs to investigate protein-protein interactions, ligand binding and other properties to evaluate their potential as pharmaceutical agents [1,2]. This is the case of amyloidogenic proteins involved in several degenerative diseases such as Alzheimer’s, Parkinson’s, and Huntington’s diseases, spongiform encephalopathies and familial amyloidotic polyneuropathies [3].

Human transthyretin (hTTR) is a 55 kDa homotetrameric serum protein with a main role in transporting thyroid hormones (T4 and T3) and retinol through binding to the retinol-binding protein (RBP) [4,5]. It is involved in pathologies such as senile systemic amyloidosis (SSA) and familial amyloidotic polyneuropathy (FAP), the latter being an autosomal dominant lethal disease in which amyloid fibrils are mostly constituted by mutant TTR variants [6,7,8], TTR cardiac amyloidosis [9] and other TTR-related cerebral amyloidosis [10]. One of the therapeutic strategies to ameliorate the progress of TTR-associated amyloidosis is the stabilization of the soluble tetrameric form of the protein to prevent aggregation and fibril formation [11,12,13]. Several screening programs have evaluated different classes of small-molecule ligands able to inhibit the amyloidogenic process at some stage. To date, only two small molecules, the orphan drug Tafamidis [14] and the NSAID diflunisal [15,16], have obtained approval for clinical use [17]. Tafamidis (Vyndaqel, Pfizer Inc., New York, NY, USA) is the only TTR tetramer stabilizer approved for use in patients with TTR polyneuropathy [18] and in patients with transthyretin amyloid cardiomyopathy [19,20]. The drug Tolcapone (registered as TASMAR), an orally active catechol-*O*-methyltransferase (COMT) inhibitor approved for Parkinson’s disease, is also an efficient TTR tetramer stabilizer and has been repurposed for FAP [21,22] and for cerebral amyloidosis [23]. Recently, the small-molecule AG10, a potent and highly selective TTR stabilizer that was designed to mimic the structural influence of the protective T119M mutation [24] is now in phase 3 trials [25]. Another interesting molecule is the palindromic molecule mds84 now in preclinical development [26].

hTTR has also shown to have a neuroprotective role in Alzheimer disease (AD). AD is a multifactorial neurodegenerative disorder [27] characterized by the formation and deposition of amyloid-β peptide (Aβ), the formation of neurofibrillary tangles in the brain [28,29], and by synaptic dysfunction accompanied with neuroinflammation [30,31]. TTR is the major Aβ binding protein in human cerebrospinal fluid (CSF) [32] that prevents Aβ aggregation with consequent abrogation of toxicity [33,34,35,36].

Studies aimed at finding TTR ligands acting as amyloidogenesis inhibitors for therapeutic intervention in TTR-associated amyloidotic polyneuropathies or acting as molecular chaperones to enhance the TTR:Aβ interaction and improve the neuroprotective effect in AD do require large amounts of hTTR protein to conduct in vitro screening assays. TTR expression in recombinant *Eschericchia coli* is often hampered by low production yields. Few studies have reported to achieve high cell density cultures with moderate recombinant TTR productivity. The first protocols produced secreted protein into the culture medium to facilitate purification but with low production yields, in the range of 5 mg/L in flask cultures [37,38]. Intracellular expression improved production yields of purified protein after two chromatographic steps to 60 mg/L of culture. Other protocols were aimed at improving the purification process by the addition of tag extensions for affinity chromatography in the purification protocol with yields of 130 mg/L of culture [39], but the additional enzymatic steps to release the native protein lowered productivity and raised production costs [40]. We reported the production of native human TTR where a single *N*-terminus methionine was added for recombinant expression in *E. coli* cells as compared to the native sequence of mature human TTR after signal peptide processing with production yields of purified protein in the range of 150 mg/L [41]. Here, we optimize the production strategy initially developed in flask cultures and adapt it to fed-batch bioreactor production.

## 2. Results and Discussion

### 2.1. Culture Medium and Induction Conditions

The gene coding for human TTR was cloned in a pET38b vector for high protein expression where no tags and just an *N*-terminus methionine was added in order to express the closest form to mature human TTR [41]. Here, we analyze the expression and purification of wt TTR and the highly amyloidogenic Y78F mutant with the objective of defining a standardized protocol for high protein expression and purification of homogeneous protein with >98% purity.

**Figure 1:**
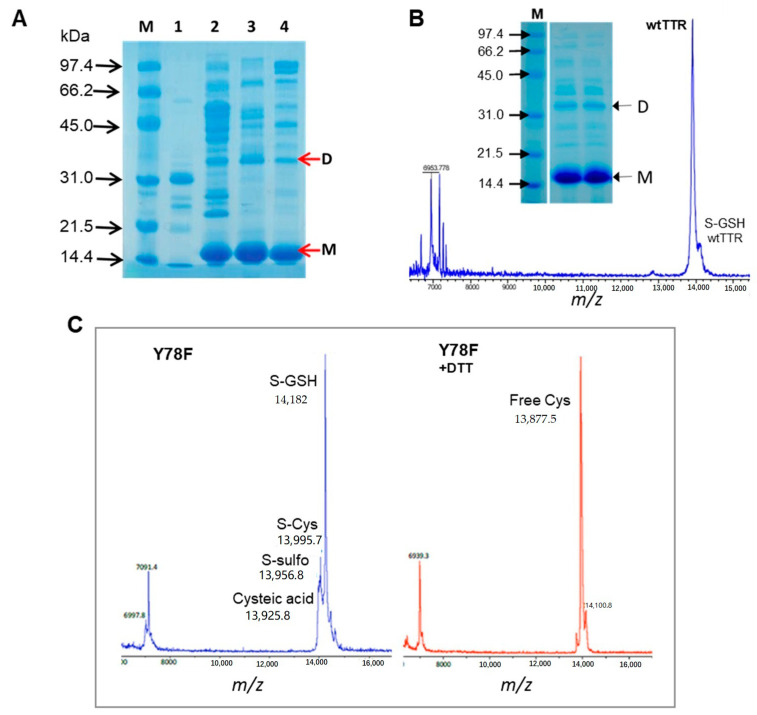
(**A**) Induction time for transthyretin (TTR) expression in flask cultures. SDS-PAGE of protein fractions after partial purification by anion exchange chromatography from cultures induced with IPTG. Lanes: M: molecular weight marker, 1. no IPTG induction (no TTR expression), 2. Induction at late exponential phase, 3. mid exponential phase, 4, beginning of the exponential growth. M: monomer, D: dimer. (**B**) MALDI-TOF MS and SDS-PAGE of purified wtTTR. (**C**) MALDI-TOF MS of Y78F TTR (left) and reduced Y78F TTR (right).

Our initial protocol for recombinant expression in flask cultures used rich Luria broth (LB) or 2× YT medium supplemented with kanamycin and IPTG induction at 37 °C [41]. When modifying the inductor concentration and post-induction temperature, best results in terms of final TTR yield and reproducibility were obtained when the culture medium was inoculated with an overnight culture of fresh *E. coli* transformants and cells were grown at 37 °C until an OD600 of 0.6 before induction (Figure 1A). IPTG induction at final concentrations of 0.3 or 1 mM gave essentially the same results but lowering the post-induction temperature to 30 °C and extending the growth up to 14–16 h gave the best results. The harvested cells by centrifugation were directly used for protein purification (see below). After purification, average protein yields were in the range of 60 to 200 mg/L of culture (wt, Y78F and other mutant variants).

### 2.2. Production in Fed-Batch Bioreactor

To adapt the protocol for expression in a bioreactor, an optimized defined medium (DM) for *E. coli* BL21(DE3) cells was used [42]. A fed-batch strategy in a 10 L bioreactor was optimized. The first strategy consisted of an initial batch growth at 37 °C in DM supplemented with 25 g/L glucose until glucose was consumed (about 6 to 8 h incubation) followed by a fed-batch step for 7 h at 37 °C with glucose in DM medium. Feeding was controlled by the level of dissolved oxygen (dO2) to keep it at 5–10%, and the pH was maintained constant at 7.0 by addition of ammonium hydroxide. Induction by IPTG was performed stepwise, with a first addition to 0.4 mM IPTG after 2 h from the beginning of the fed-batch, and two subsequent additions at 3 and 5 h to reach a total added concentration of 1 mM. Under these conditions, the cultures reached an OD of 30, and the production of isolated protein after purification was about 500 mg/L of culture.

Higher cell densities in the fed-batch step were obtained with an enriched feeding medium composed of DM with 25 g/L glucose supplemented with tryptone (8 g/L) and yeast extract (5 g/L). The fed-batch started when the initial glucose was consumed and IPTG at a final 1 mM concentration was added at the beginning of the fed-batch in a single addition. The fed-batch lasted 4.5 h before harvesting the cells. After purification (see below), the final production yield was 660 mg/L of culture for the Y78F mutant hTTR. The final protocol described in the Materials and Methods section was reproducible for the wt and different TTR mutants, with average production yield of purified proteins in the range of 200–660 mg/L of culture, depending on the protein variant. This is the highest reported production yield for recombinantly expressed hTTR enabling the application of screening protocols that demand high amounts of protein.

### 2.3. Protein Purification

The cells harvested by centrifugation (either from flask or bioreactor cultures) were lysed by sonication or with a cell homogenizer (in the case of large cultures). After centrifugation, the supernatant was subjected to fractional ammonium sulfate precipitation (40, 55 and 85% (NH_4_)_2_SO_4_). TTR precipitated in the third fractions (between 55 and 85%). The precipitate was dissolved in Tris buffer and dialyzed against the same buffer. The first chromatographic step was anion exchange chromatography with Q-Sepharose at pH 7.6. At this high pH, about 2 pH units above the TTR isoelectric point (calculated pI of 5.4), the pH may vary from 7.2 to 8.0 with no effect on final protein yields. In the standardized protocol (see methods) TTR was eluted with a gradient from 0.1 to 0.5 M NaCl in Tris buffer pH 7.6. The combined TTR-containing fractions were extensively dialyzed and lyophilized. The lyophilization step was introduced after checking that it did not affect the properties of the final protein (solubility, amyloidogenic behavior) since it simplifies the concentration of the diluted protein before gel filtration chromatography. The lyophilized protein was dissolved in a small volume of Tris buffer pH 7.6, 0.1 M NaCl and loaded into a Superdex 75 gel filtration column. The eluted protein was dialyzed against water and lyophilized for storage and dissolved in the corresponding buffer before use.

### 2.4. Protein Quality Assessment

The quality and performance of the different protein batches (wt and Y78F mutant hTTR) was assessed by SDS-PAGE electrophoresis, MALDI-TOF mass spectrometry, and a fibrillogenesis assay.

Native TTR is an homotetramer that appears as a dissociated monomer with traces of dimer under standard SDS-PAGE conditions with a loading buffer containing 2% (*w*/*v*) SDS and heating the samples at 100 °C for 2 min. An example of purified protein after the expression and purification protocol is shown in Figure 1B for wt TTR.

Native hTTR occurs in vivo as a very heterogeneous protein due to post-translational modifications (PTM) at Cys-10, the single Cys residue in the protein sequence. It has been reported that only around 10–15% of the circulating TTR in plasma remains unmodified at Cys-10, finding S-sulfonation (*S*-Sulfo), *S*-glycinylcysteinylation (*S*-CysGly), *S*-cysteinylation (*S*-Cys) and *S*-glutathionylation (*S*-GSH) as the most prevalent forms [43,44,45]. Despite carrying out protein expression in *E. coli*, the produced proteins, both wt and Y78F mutant, were modified at Cys-10. Figure 1B,C show two examples of MALDI-TOF mass spectra of representative batches of wt and Y78F hTTR. The wt protein consistently gives the unmodified form as the main component with minor glutathionated protein (*S*-GSH). However, the Y78F mutant produces mainly the glutathionated form and the *S*-Cys, *S*-Sulfo and cysteic acid forms as minor components. Treatment of Y78F hTTR with DTT reduces the *S*-GSH form to free TTR as shown in Figure 1C.

**Figure 2:**
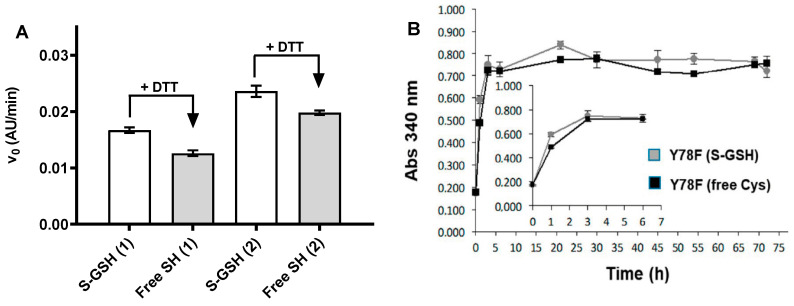
Acid-induced fibrillogenesis of Y78F TTR isoforms *S*-GSH (glutathionated) and free Cys (reduced with DTT). (**A**) Initial rate of acid-induced fibrillogenesis determined for two different protein batches (1 and 2). (**B**) Absorbance monitoring for 72 h. Inset. Magnification of the initial region.

The amyloidogenic capacity is used as a quality parameter of the different protein batches. In vitro assays with purified TTR (wt or clinically relevant TTR mutants) monitor acid-induced aggregation and fibrillation, commonly by lowering the pH to 4.2–4.4 for wt and low amyloidogenic mutants, or higher pH values (6–7) for highly amyloidogenic mutants. These methods include turbidimetric assays and thioflavin T binding assays (reviewed in [46]). Here, acid-induced aggregation was evaluated by a turbidimetric assay monitoring changes in absorbance at 340 nm [41]. The fibrillogenesis capacity is quantified by the initial rate of aggregate/fibril formation in a short-term assay (1.5 h), whereas an end-point assay evaluates the total aggregate formation after long incubations (72 h) at acidic pH. By initial rates, two different protein batches of Y78F hTTR are compared in Figure 2A. The variation between batches is about 15%, and the glutathionated (*S*-GSH) protein is about 1.25-fold more amyloidogenic than the reduced protein (free SH). When fibrillogenesis of *S*-GSH and reduced proteins was monitored up to 72 h, both protein forms reach the same amount of final aggregates/fibrils (Figure 2B). In conclusion, recombinant hTTR expressed and purified according to the protocol here described has the required quality for its use in fibrillogenesis assays with reproducible results with variations in the initial aggregation rate not larger than 15% between different protein batches, as assessed from more than 10 productions used in screening campaigns.

### 2.5. Applications to Screening Assays

The high expression yields of recombinant hTTR, either wt, highly amyloidogenic variants or clinically relevant mutants obtained with our optimized protocol (200–660 mg/L of culture depending on the mutant) as homogeneous proteins have enabled their application in screening programs for the evaluation of libraries of TTR amyloidogenesis inhibitors as well as the evaluation of small-molecule chaperones enhancing the interaction between TTR and Amyloid-β (Aβ) peptides.

#### 2.5.1. Kinetic Turbidimetry Assay for Screening TTR-Amyloidogenesis Inhibitors

**Figure 3:**
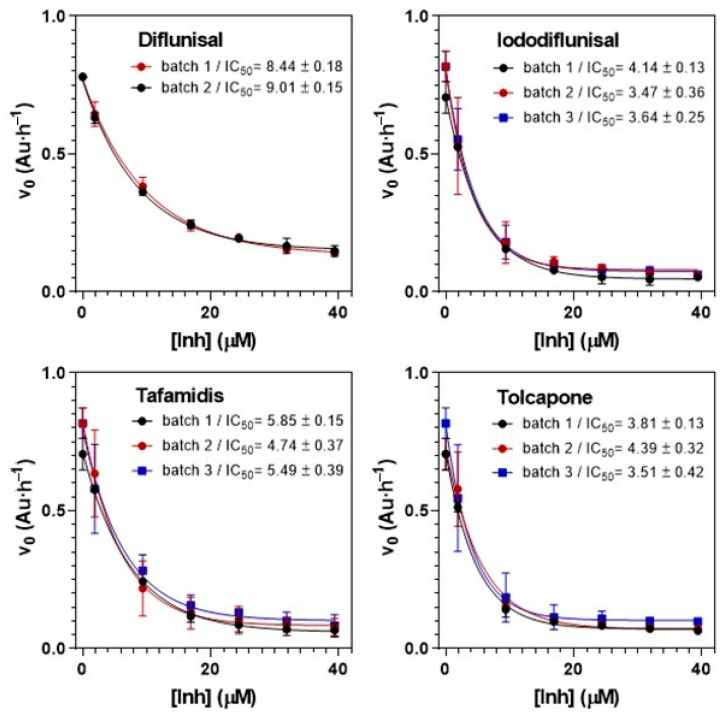
TTR fibrillogenesis inhibitors. Reproducibility of IC_50_ (μM) determination with different TTR Y78F protein batches. Initial rate of acid-induced fibrillogenesis at increasing concentrations of inhibitor.

Based on the acid-induced properties of hTTR, this assay evaluates the ability of small molecules to inhibit fibrillogenesis/aggregation in a dose-dependent manner to quantify two relevant inhibition parameters: IC_50_, concentration of inhibitor at which the initial rate of fibril formation is one-half than that without inhibitor, and RA(%), percent reduction of fibril formation rate at high inhibitor concentration relative to the rate in the absence of inhibitor. The initial protocol reported in [41] has been extensively applied over the years [47,48,49,50]. Key for comparative evaluation of inhibitors from different screening campaigns is the homogeneity and quality of the recombinant hTTR used. Figure 3 illustrates selected examples of TTR tetramer stabilizers (diflunisal, iododiflunisal, tafamidis and tolcapone). The optimized assay protocol (see Section 3.6) has been shown to provide reproducible results with coefficients of variation (CV) ≤ 10% for IC_50_ values in the range of 3–15 μM and for RA(%) values in the range of 60–100% when using different protein batch productions.

#### 2.5.2. Turbidity-Based Assay for Screening Small-Molecule Chaperones Enhancing the TTR:Aβ Interaction

**Figure 4:**
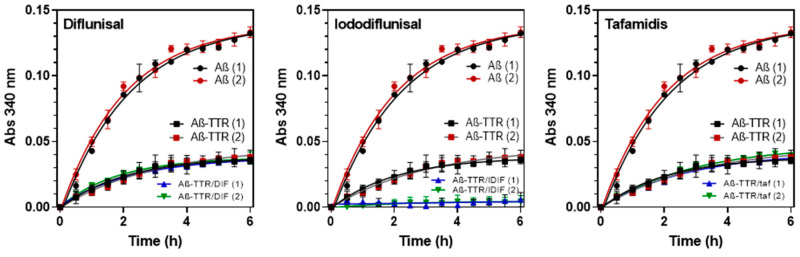
Small-molecule chaperones of TTR:Aβ interaction. Reproducibility with two different wt TTR protein batches (1, 2). % reduction of aggregates formation (RA%) relative to Aβ(12–28) after 6 h incubation: Aβ(12–28) + TTR = 78.1 ± 2.9; Aβ(12–28) + (TTR + DIF) = 78.3 ± 2.5; Aβ(12–28) + (TTR + IDIF) = 96.5 ± 3.1; Aβ(12–28) + (TTR + tafamidis) = 77.5 ± 1.7.

Inspired by the previous assay, we developed a rapid and simple high-throughput assay to screen for small-molecules that may act as chaperones of the TTR:Aβ interaction. The assay monitors Aβ(12-28) aggregation in the presence of both wt TTR and a small molecule compound (SMC). The small-molecule chaperones form ternary soluble complexes TTR/Aβ/SMC that prevent Aβ aggregation. The method uses the shorter Aβ(12-28) sequence, a cheaper model system for aggregation by Aβ(1-40). The test is carried out in 96-well plates that are UV monitored for turbidity for 6 h. Figure 4 shows selected examples with TTR ligands (diflunisal, iododiflunisal and tafamidis). The aggregation kinetics can be monitored with parallel results by other assays as thioflavin T (ThT) fluorescence assays of the amyloid structures formed [36].

## 3. Materials and Methods

### 3.1. Protein Expression

Recombinant wt and Y78F mutant TTR were expressed from phTTR.wt-I and phTTR.Y78F-I plasmids based on a pET38b(+) (Novagene, Merk Biosciences, Darmstadt, Germany) vector as reported [41]. Both wt and Y78F TTR proteins were expressed in *E. coli* BL21(DE3) cells harboring the corresponding plasmids with kanamycin resistance as selection marker. Final protocols for protein expression were:

#### 3.1.1. Batch Expression in Flask Cultures

A total of 2 mL LB (or 2× YT) medium supplemented with 100 μg/mL kanamycin (Kan) was inoculated with a single colony from an LB-agar plate of freshly transformed *E. coli* cells and grown overnight at 37 °C. 100 μL of the initial culture were used to inoculate 50 mL of 2xYT+ 100 μg/mL Kan at 37 °C until an optical density (OD600) of ~4. Finally, 1.3 L of 2xYT+ 100 μg/mL Kan were inoculated with 5 mL of the previous culture in a 2 L Erlenmeyer flash with deflectors for improved aeration. Cells were grown at 37 °C with shaking at 200× rpm until an OD600 of 0.6 (about 6 h). IPTG was added to a final 1 mM concentration and the culture incubated at 30 °C for 14–16 h. Cell were harvested by centrifugation at 4 °C, 10,000× rpm for 10 min and directly used for protein purification.

#### 3.1.2. Expression in Fed-Batch Bioreactor

The defined medium (DM) is composed of 13.23 g/L K_2_HPO_4_, 2.65 g/L KH_2_PO_4_, 2.04 g/L NaCl, 4.1 g/L (NH_4_)SO_4_, 0.026 g/L FeCl_3_, 0.5 g/L MgSO_4_·7H_2_O, 0.01 g/L thiamine, 25 g/L glucose and 2.86 mL/L Trace Elements Solution composed of 0.04 g/L AlCl_3_·6H2O, 0.16 g/L CoCl_2_·6 H_2_O, 0.01 g/L H_3_BO_3_, 0.01 g/L NiCl_2_·7H2O, 0.87 g/L ZnSO_4_·7H_2_O, 1.55 g/L CuSO_4_·5H_2_O, 1.42 g/L MnCl_2_·4H_2_O, 0.02 g/L NaMoO_4_, 1.44 CaCl_2_·2H_2_O. A 10 L bioreactor (Applikon Biotechnology, Delft, The Netherlands) containing 4 L of DM supplemented with 100 mg/L Kan was inoculated with 50 mL of freshly prepared culture in DM medium at OD600 of 0.6. The batch culture was grown at 37 °C with increasing agitation (300 to 1000 rpm) to maintain the dO_2_ at 5–10%. When glucose was consumed and the dissolved oxygen increased, the fed-batch was initiated with a feeding solution of DM supplemented with 100 mg/L kanamycin, 8 g/L tryptone and 5 g/L yeast extract, the temperature was set at 30 °C and the culture induced with IPTG (1 mM final concentration) at the beginning of the fed-batch step. During all the culture the pH was maintained at 7.0 with NH_4_OH (30% stock solution). During the fed-batch, agitation was maintained at 1000 rpm, and pO_2_ at 5–10% by supplying additional oxygen if dO_2_ < 5% or adding feeding solution if dO_2_ > 5%. At 4.5 h after IPTG induction, cells were harvested by centrifugation at 4 °C, 10,000 rpm for 15 min and the pellet directly used for protein purification.

### 3.2. Protein Purification

All volumes in the purification steps are given for processing the cell pellet coming from 1L culture. The cell pellet was resuspended in 250 mL lysis buffer (0.5 M Tris-HCl, 1 mM PMSF, pH 7.6), and lysed with a cell disrupter at 20 kpsi (Constant Systems Ltd., Daventry, Northants, UK). Viscosity of the homogenate was reduced by applying short sonication cycles before centrifugation at 4 °C, 12,000× rpm for 30 min to collect the supernatant.

(i) Ammonium sulphate precipitation. Three precipitation steps at 4°C were performed by addition of (NH_4_)_2_SO_4_ up to 40, 55 and 85%. The precipitate fraction after the last step (55 to 85% fraction) contained most of the TTR protein (as judged by SDS-PAGE). The precipitate was resuspended in 50 mL of buffer A (20 mM Tris·HCl, 0.1 M NaCl, pH 7.6), and dialyzed (3×) against buffer A at 4 °C using a MWCO 6–8 kDa, 40 mm Cellu.Sep Regenerated Cellulose Tubular Membranes (Membrane Filtration Products, Inc., Seguin, TX, USA).

(ii) Anion exchange chromatography. The dialyzed solution was loaded to a XK 26/40 column (120 mL) with Q-Sepharose High Performance resin using an ÄKTA FPLC System (Cytiva, Marlborough, MA, USA). Buffer A was used for column conditioning (at 1 mL/min) and protein loading (at 2 mL/min). Elution (1 mL/min) was done with a NaCl linear gradient (0.1 M to 0.5 M NaCl in Buffer A). Fractions were collected and analyzed by SDS-PAGE. All fractions containing TTR were combined and dialyzed against deionized water at 4 °C (MWCO 6–8 kDa) three times for 8 h. The final solution was lyophilized.

(iii) Gel filtration chromatography. The lyophilized sample was dissolved in 4 mL of Buffer A and loaded at 2 mL/min flow rate onto a XK 26/100 column (440 mL) with Superdex 75 prep Grade resin using an ÄKTA FPLC System (GE Healthcare Life Sciences). Protein elution was performed with Buffer A at 0.3 mL/min and eluted fractions were collected and analyzed by SDS-PAGE. Fractions containing TTR were combined and dialyzed against deionized water at 4 °C (MWCO 6–8 kDa, 3× for 8 h each) and lyophilized for storage at −20 °C.

### 3.3. Reduction with 1,4-Dithiothreitol (DTT)

A 4 mg/mL TTR solution in 20 mM Tris·HCl, 0.1 M NaCl, pH 7.6 was treated with 1 mM DTT during 1h at room temperature. Completion of the reduction reaction was checked by MALDI-TOF MS. The reduced solution was dialyzed against 20 mM K_2_HPO_4_, 0.1 M KCl, pH 7.6 buffer at 4 °C.

### 3.4. MALDI-TOF Mass Spectrometry of Purified Proteins

A saturated solution of sinapinic acid (SA) as matrix in 30:70 (*v*/*v*) acetonitrile:water, 0.1% trifluoroacetic acid (TFA) and a protein solution in 30:70 (*v*/*v*) acetonitrile:water, 0.1% TFA were prepared and mixed in a 1:1 ratio. A total of 1 µL of the previous mixture was deposited into a polished stainless-steel target (Bruker, Bremen, Germany) and allowed to dry. Then, 1 µL of SA matrix solution was deposited into the sample and allowed to dry. The same procedure was followed for the Protein Standard Calibration I solution (Bruker) used for Calibration. The target was introduced in a Autoflex MALDI-TOF (Bruker), spectra were acquired in lineal mode (Flex control, Bruker) and processed by Flex Analysis (Bruker).

### 3.5. Acid-Induced TTR Fibrillogenesis Assay

TTR stock solution: 4 mg/mL in 20 mM phosphate, 100 mM KCl, pH 7.6. Incubation buffer: 10 mM phosphate, 100 mM KCl, 1 mM EDTA, pH 7.6. Dilution buffer: 400 mM sodium acetate, 100 mM KCl, 1 mM EDTA, pH 4.2. The assay was performed in 96-well microplates. 20 μL of TTR stock solution were added into each well. The final DMSO content of each well was adjusted up to 5% (final assay concentration) with DMSO:H_2_O (1:1). Incubation buffer was added up to a volume of 100 μL. The 96-well plate was introduced into the thermostated microplate reader for 30 min at 37 °C, with orbital shaking for 15 s every min. Fibril formation was then induced by addition of 100 μL of dilution buffer to each well. The 96-well plate was placed again into the microplate reader and incubated at 37 °C with shaking (15 s every minute) during 1.5 h. Absorbance at 340 nm was monitored at each minute. All assays were run in triplicate.

For end-point assays, the same procedure was performed in sealed HPLC vials in order to avoid evaporation. A total of 40 μL of TTR stock solution was added to each vial. The final DMSO content was then adjusted up to 5% with DMSO:H_2_O (1:1). Incubation buffer was added up to a volume of 200 μL. The vials were sealed and incubated at 37 °C for 30 min. Afterwards, fibril formation was induced by addition of 200 μL of dilution buffer to each vial. After soft mixing, 200 μL of each vial were placed into a well in a 96-well plate and absorbance at 340 nm was measured (starting point, *t* = 0). After measurement, the 200 μL were placed back into its corresponding HPLC vial, which was sealed again and placed at 37 °C. Measurements were performed at 1, 3, 6, 21, 30, 45, 54, 69 and 72 h following the same procedure. All assays were run per duplicate.

### 3.6. Kinetic Turbidimetry Assay for Screening TTR Fibrillogenesis Inhibitors

Protein stock solution, incubation buffer and dilution buffer were prepared as above. Protocol for one inhibitor: 20 μL of hTTR Y78F stock was dispensed into seven wells of a 96-well microplate. A total of 20 μL of inhibitor solutions in DMSO:H_2_O (1:1) was added to give final concentrations ranging from 0 to 40 μM and DMSO final content of 5% (in the final 200 μL assay). Incubation buffer (60 μL) was then added up to a volume of 100 μL. The plate was incubated at 37 °C in a thermostated microplate reader with orbital shaking 15 s every minute for 30 min. 100 μL of dilution buffer were dispensed to each well, and the mixture was incubated at 37 °C with shaking (15 s every min) in the microplate reader. Absorbance at 340 nm was monitored for 1.5 h at 1 min intervals. Data were collected and analyzed using Microsoft Excel software. All assays were done in duplicate. From the initial rates (Abs vs. time) at different inhibitor concentrations, the inhibition parameters IC_50_ and RA (%) were calculated as reported [41,47,48,49,50,51].

### 3.7. Turbidimetry Assay for Screening Small-Molecule Chaperones of TTR:Aβ Interaction

The following stock solutions were used: Buffer A: 25 mM HEPES buffer, 10 mM glycine, pH 7.4. Protein (wt hTTR) stock: 9.5 mg/mL (170 µM) in 25 mM HEPES buffer, 10 mM glycine, pH 7.4 and 5% DMSO (final concentration) was prepared in the absence of salt (buffer A). Aβ(12–28) stock: 0.4 mg/mL (200 µM) in 25 mM HEPES buffer, 10 mM glycine, pH 7.4 and 5% DMSO (final concentration). For the small-molecule compound, a first 10 mM solution in DMSO was prepared and the final stock was prepared by mixing 50 µL of the previous DMSO solution with 950 µL of buffer A (final 5% DMSO). First, the small-molecule compound and TTR complex was formed. To this end, 60 µL of TTR stock was dispensed into the wells of a 96-well microplate. A total of 40 µL of small-molecule stock was added to give final concentrations of 100 µM. The plate was introduced in the thermostated microplate reader (SpectraMax M5 Multi-Mode Microplate Readers, Molecular Devices Corporation, Sunnyvale, CA, USA) and incubated for 1 h at 37 °C with orbital shaking 15 s every 30 min. Then, 100 μL of Aβ solution was added to the well to give 100 μM ligand:50 μM TTR:100 μM Aβ final concentrations. The plate was incubated at 37 °C in the microplate reader with orbital shaking 15 s every minute for 30 min. The absorbance at 340 nm was monitored for 6 h at 30 min intervals. Data were collected and analyzed using MS Excel software (Microsoft, Redmond, WA, USA). All assays were done in duplicate. From the different absorbance readings between sample and controls, the percent reduction of aggregates formation (RA%) in the presence and absence the small-molecule compound/TTR complex were determined.

## 4. Conclusions

The optimized expression and purification protocol for recombinant hTTR production (wt and amyloidogenic mutants) yields up to 660 mg of homogenous protein per L of culture in fed-batch bioreactor. Whereas the recombinant wt protein is mainly unmodified at Cys10, the highly amyloidogenic Y78F variant renders mainly the *S*-glutathionated form. When comparing the *S*-glutathionated and reduced Y78F proteins, amyloidogenicity (as determined by a turbidimetry assay) is little affected by this post-translational modification. The inter-batch reproducibility of expression and protein quality has allowed the implementation of screening assays for the evaluation of libraries of TTR amyloidogenesis inhibitors, as well as the evaluation of small-molecule chaperones of the TTR:Aβ interaction.
